# Does Type 2 Diabetes Mellitus Increase Postoperative Complications in Patients Submitted to Cardiovascular Surgeries?

**DOI:** 10.21470/1678-9741-2019-0027

**Published:** 2020

**Authors:** Cauê Padovani, Regiane Maria da Costa Arruda, Luciana Maria Malosá Sampaio

**Affiliations:** 1 Universidade Nove de Julho - UNINOVE, Campus Vergueiro, São Paulo, SP, Brazil.

**Keywords:** Diabetes Mellitus, Cardiovascular Diseases, Cardiovascular Surgical Procedures, Postoperative Complications, Intensive Care Units

## Abstract

**Objective:**

To compare the incidence of postoperative complications (PC) between diabetic and nondiabetic patients undergoing cardiovascular surgeries (CS).

**Methods:**

This is a retrospective cross-sectional study, based on the analysis of 288 medical records. Patients aged ≥ 18 years, admitted to the intensive care unit (ICU) between January 2012 and January 2013, and undergoing coronary artery bypass grafting (CABG) or vascular surgeries were included. The population was divided into those with and without type 2 diabetes mellitus (T2DM), and then it was evaluated the incidence of PC between the groups.

**Results:**

The sample included 288 patients, most of them being elderly (67 [60-75] years old) male (64%) subjects. Regarding to surgical procedures, 60.4% of them were undergoing vascular surgeries and 39.6% were in the postoperative period of CABG. The incidence of T2DM in this population was 40% (115), just behind hypertension, with 72% (208). Other risk factors were also observed, such as smoking in 95 (33%) patients, dyslipidemias in 54 (19%) patients, and previous myocardial infarction in 55 (19%) patients. No significant difference in relation to PC (bleeding, atrial fibrillation, cardiorespiratory arrest, and respiratory complications) between the groups was observed (*P*>0.05).

**Conclusion:**

T2DM has a high incidence rate in the population of critically ill patients submitted to CS, especially in the elderly. However, in this small retrospectively analyzed study, there was no significant increase in PC related to diabetes for patients undergoing CS.

**Table:** 

Abbreviations, acronyms & symbols	
ADA	= American Diabetes Association
CABG	= Coronary artery bypass grafting
CS	= Cardiovascular surgeries
HbA1c	= Glycated hemoglobin
ICU	= Intensive care unit
IQ	= Interquartile range
LDL	= Low-density lipoprotein
PC	= Postoperative complications
SPSS	= Statistical Package for the Social Sciences
T2DM	= Type 2 diabetes mellitus
WHO	= World Health Organization

## INTRODUCTION

Diabetes is a complex disease that affects millions of people worldwide. The diabetes rate has increased in recent years, with the greatest increment in middle and low-income countries^[1,2]^. The World Health Organization (WHO) estimates 439 million adults with diabetes for 2030. According to WHO, about 16 million Brazilians suffer from diabetes. This disease incidence rate has grown 61.8% in the last ten years and Brazil occupies the 4^th^ place in the ranking of countries with the highest number of diabetes cases, behind China, India, and the United States of America^[3]^.

Hyperglycemia is the major risk factor for complications in patients with type 2 diabetes mellitus (T2DM)^[4]^. Hyperglycemia causes glycation of tissues, which almost inevitably leads to acute disturbances in metabolism and long-term end-organ damage, especially in the blood vessels, heart, and nerves. Therefore, T2DM can lead to numerous complications, such as retinopathy, renal dysfunction, neuropathies, microangiopathies, amputations, myocardial infarction, and stroke^[1,2]^. In patients with T2DM, a glycated hemoglobin (HbA1c) level outside the target range was the strongest predictor of stroke and acute myocardial infarction^[5]^. T2DM presents a two to fourfold increase in risk of incident coronary heart disease and ischemic stroke and a 1.5 to 3.6-fold increase in mortality^[1,2]^.

Studies have shown that T2DM is associated with increased cardiovascular morbidity and mortality, as well as the need for surgical procedures and hospitalizations in the intensive care unit (ICU). Some reports indicate that diabetic patients present greater morbidity, such as prolonged hospitalization, infections, respiratory failure, and renal and cerebral complications^[2,4-6]^. On the other hand, some studies have shown that there was no association of T2DM with increase in the incidence of postoperative complications^[7]^ or risk for in-hospital mortality^[8,9]^. A broad consensus has not been reached because these findings were not uniformly confirmed by records and other studies^[7-10]^.

Thus, the aim of the present study was to compare the incidence of postoperative complications between diabetic and nondiabetic patients undergoing cardiovascular surgeries.

## METHODS

### Study Design

This is a retrospective cross-sectional study, based on the analysis of 288 medical records. The present study was conducted in a tertiary public hospital in the city of São Paulo. It was developed with the consent of the participants and/or their families, in accordance with Resolution 196/96 of the National Health Council. The research was approved by the Ethics and Research Committee of the Municipal Health Department of São Paulo - SMS/SP (number 263,790).

### Study Population and Variables

Patients were included in this study if they were ≥ 18 years old, admitted to the ICU between January 2012 and January 2013, and undergoing coronary artery bypass grafting (CABG) or vascular surgeries.

Patients’ demographic and clinical data included age, gender, type of surgery, postoperative complications, use of vasoactive drugs, personal history, and comorbidities. Data were collected from patients' records.

Diabetes mellitus diagnosis was performed according to the American Diabetes Association (ADA) guidelines^[11]^: HbA1c ≥ 6.5%, or fasting serum glycemia ≥ 126 mg/dL, or serum glycemia ≥ 200 mg/dL after ingestion of 75 g of glucose, or random serum glycemia ≥ 200 mg/dL accompanied by symptoms attributed to hyperglycemia.

The patients were then divided into those with and without T2DM.

### Statistical Analysis

All statistical analyses were performed using the Statistical Package for the Social Sciences (SPSS) software (SPSS, Microsoft Inc., Chicago, Il, USA), version 20.0. The Shapiro-Wilk test was used to evaluate the normality of the data. The descriptive analysis for qualitative variables was performed from the distribution of absolute and relative frequency (%), and the quantitative variables were presented as median (interquartile range 25% to 75%). The comparison between groups was performed by Mann-Whitney U test for quantitative variables and by Chi-Square test for categorical variables. The significance level used for the tests was 5%.

## RESULTS

The sample included 288 patients, most of them being elderly male subjects. The demographic characteristics are described in Table 1. No significant difference in variables between the two groups was observed.

**Table 1:** Demographic baseline characteristics of diabetic and nondiabetic patients.

Variables	Diabetic (n=100)	Nondiabetic (n=188)	Total (n=288)	*P*-value
Gender, male, n (%)	63 (63)	120 (64)	184 (64)	0.866
Age (years), median (IQ 25% to 75%)	66 (61-74)	68 (59-75)	67 (60-75)	0.843
Length of ICU stay (days), median (IQ 25% to 75%)	4 (3-6)	3 (3-5)	4 (3-5)	0.075

ICU=intensive care unit; IQ=interquartile range

**Fig. 1:**
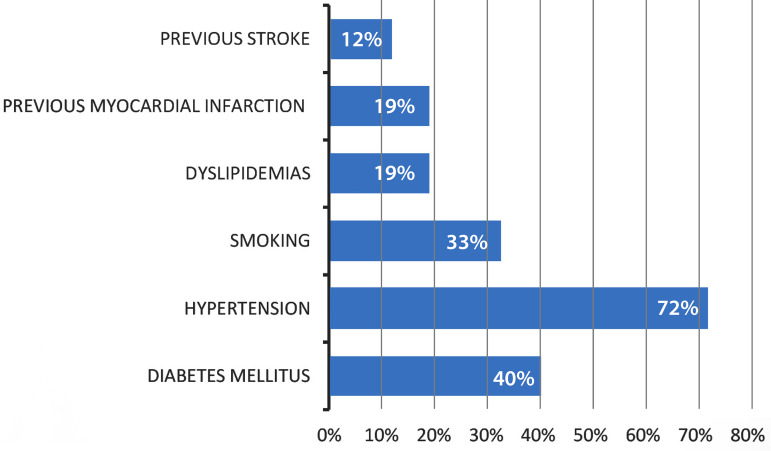
Incidence of diabetes mellitus and other cardiovascular risk factors.

The incidence of T2DM in this population was 40% (115), just behind hypertension, with 72% (208). Other risk factors such as smoking, dyslipidemias, previous myocardial infarction, and previous stroke were also observed. The incidence of diabetes mellitus and other cardiovascular risk factors are shown in Figure 1. Regarding to surgical procedures, 60.4% of the patients were undergoing vascular surgeries and 39.6% were in the postoperative period of CABG. The list of procedures performed is described in Table 2.

**Table 2:** Surgical procedures performed (n=288).

Surgeries	Values
Coronary artery bypass grafting, n (%)	114 (39.6)
Vascular surgeries, n (%)	174 (60.4)
Angioplasty and grafting of lower limbs, n (%)	56 (19.4)
Correction of aortic or lower limb aneurysm, n (%)	42 (14.6)
Carotid endarterectomy, n (%)	31 (10.8)
Lower limb amputation, n (%)	20 (6.9)
Thromboembolectomy of lower limbs, n (%)	16 (5.6)
Others, n (%)	9 (3.1)

Additionally, the vast majority of patients used vasoactive drugs in the postoperative period of cardiovascular surgeries. The use and types of vasoactive drugs are described in Table 3.

**Table 3:** Vasoactive drugs (n=288).

Variables	Values
Use of vasoactive drugs, yes, n (%)	273 (95)
Types of vasoactive drugs	
Dobutamine, n (%)	242 (84)
Noradrenaline, n (%)	118 (41)
Sodium nitroprusside, n (%)	49 (17)
Nitroglycerin, n (%)	46 (16)

Regarding to operative morbidity, we highlight bleeding, atrial fibrillation, cardiorespiratory arrest, and respiratory complications. No significant difference in relation to postoperative complications between the two groups was observed. Comparisons between diabetic and nondiabetic patients for the occurrence of postoperative complications are described in Table 4.

**Table 4:** Comparison between diabetic and nondiabetic patients for the occurrence of postoperative complications.

Postoperative complication	Diabetic (n=100)	Nondiabetic (n=188)	Total (n=288)	*P*-value
Bleeding, n (%)	8 (8)	13 (6.9)	21 (7.3)	0.736
Atrial fibrillation, n (%)	5 (5)	8 (4.2)	13 (4.5)	0.772
Cardiorespiratory arrest, n (%)	4 (4)	6 (3.2)	10 (3.5)	0.721
Respiratory complications, n (%)	5 (5)	8 (4.2)	13 (4.5)	0.772

## DISCUSSION

Our results show that T2DM has a high incidence rate in the population of critically ill patients submitted to cardiovascular surgeries, especially in the elderly. However, in this relatively small series of patients, it was not observed a significant increase in postoperative complications related to diabetes in patients submitted to cardiovascular surgeries. These results are similar to those presented by other previous studies^[7,10]^.

The reported incidence of postoperative complications after cardiovascular procedures in diabetic patients has been varied. Approximately 25% of patients who undergo CABG have diabetes mellitus. In relation to surgical treatment, several studies revealed higher morbidity and perioperative mortality rates among diabetic patients undergoing CABG, as well as decreased survival rate after this procedure^[12]^. The presence of diabetes is considered to be an independent risk factor for postoperative mortality after CABG, with an odds ratio of 1.73 for cardiovascular death and 2.94 for overall mortality^[12]^. Moreover, Brazilian studies observed the association of diabetes with general infections in the postoperative period^[13]^, sternal wound infection^[14]^, and mediastinitis^[15,16]^.

On the other hand, Bardakci et al.^[9]^ did not observe the presence of diabetes as an independent risk factor for hospital mortality. Similarly, in a Brazilian study performed by Alves Júnior et al.^[8]^, including patients submitted to CABG or heart valve surgeries, diabetes was not associated with increased risk for in-hospital mortality. In the present study, there was no association of T2DM with increase in the incidence of postoperative complications. López-Rodríguez et al.^[7]^ did not observe this association as well.

In addition to hyperglycemia, diabetic patients usually present other cardiovascular risk factors, including hypertension, smoking, dyslipidemia, obesity, and previous cardiovascular events^[2]^. These findings were also shown in our study. We believe that the investigation of T2DM impact on cardiovascular events and postoperative complications is hampered by the presence of multiple cardiovascular risk factors in this population. T2DM is a complex disease that leads to continuous medical care with comprehensive, multifactorial strategies for reducing cardiovascular risk. Randomized trials investigating the effect of multifactorial cardiovascular risk-factor intervention in patients with T2DM are scarce, and contemporary studies were designed to measure the cumulative incidence of cardiovascular events among patients with various risk factors (*e.g*., hyperglycemia, hypertension, dyslipidemia, and microalbuminuria) who received intensive therapy, as compared with those who received conventional therapy^[2,17]^.

The following risk factors were considered the strongest predictors for cardiovascular events and death: HbA1c, duration of diabetes, systolic blood pressure, smoking, low physical activity, and low-density lipoprotein (LDL) cholesterol levels outside the target ranges. Most of these risk factors showed a linear association with the risk of acute myocardial infarction and stroke. Levels of HbA1c, systolic blood pressure, and LDL cholesterol that were lower than the target levels were associated with lower risks of acute myocardial infarction and stroke^[5,18]^.

In T2DM patients, hyperglycemia is the principal risk factor for microvascular complications, and a decrease in HbA1c, by whatever means, reduces the risk of eye, kidney, and nerve complications. Lower HbA1c levels than are currently recommended in guidelines were associated with a lower risk of death^[4,5,19,20]^. Our results show that T2DM has a high incidence rate in the hospitalized population undergoing cardiovascular surgery. In the same way, another study showed that there was a substantial risk of hospitalization for heart failure among patients who had high HbA1c levels. The risk of hospitalization for heart failure was marginally low at HbA1c levels < 53 mmol per mole^[5]^.

In our study, the incidence of T2DM in critically ill patients submitted to cardiovascular surgeries was 40%, just behind hypertension (72%). Hypertension is a well-established risk factor for coronary heart disease and for stroke mortality for the entire population. In both types 1 and 2 diabetes, hypertension is a major risk factor for atherosclerotic cardiovascular disease and microvascular complications. In type 1 diabetes, hypertension is often the result of underlying diabetic kidney disease. In T2DM, hypertension usually coexists with other cardiometabolic risk factors^[2,21,22]^.

LDL cholesterol is one of the most important reversible risk factors for cardiovascular morbidity and mortality. The relative risk of cardiovascular mortality for diabetic patients ranged from 2.83 to 4.46 according to the level of cholesterol. Thus, cholesterol is a strong and independent risk factor for cardiovascular mortality, which is potentiated by diabetes^[2,23]^. Another important reversible risk factor for cardiovascular disease is cigarette smoking. Compared with subjects who have never smoked, the incidence of acute myocardial infarction is increased sixfold in women and threefold in men who smoke at least 20 cigarettes per day^[2,24]^. Active smoking is associated with the highest risk of total mortality and cardiovascular events among diabetic patients, while smoking cessation is associated with a reduced risk in total mortality and cardiovascular events in diabetic patients^[25,26]^.

In this context, the primary goals of T2DM management are to improve glycemic control to prevent microvascular complications and normalize cardiovascular risk factors to reduce cardiovascular events and morbidity^[4]^. Consequently, also decreasing the need for cardiovascular surgical procedures and hospitalization in the ICU. Regarding to postoperative complications, the literature presents divergences in pointing at T2DM as an unfavorable prognostic factor in patients undergoing cardiovascular procedures.

The present study has some limitations. Firstly, this report used a retrospective method. Secondly, this study used a database from a single institution (one center). And finally, the impact of the results of this study may be limited by the sample size and might be confirmed by future randomized clinical trials.

## CONCLUSION

T2DM has a high incidence rate in the population of critically ill patients submitted to cardiovascular surgeries, especially in the elderly. However, in this small retrospectively analyzed study, there was no significant increase in postoperative complications related to diabetes for patients submitted to cardiovascular surgeries.

**Table:** 

Author's roles & responsibilities	
CP	Substantial contributions to the conception or design of the work; or the acquisition, analysis, or interpretation of data for the work; drafting the work or revising it critically for important intellectual content; final approval of the version to be published
RMCA	Drafting the work or revising it critically for important intellectual content; final approval of the version to be published
LMMS	Final approval of the version to be published
